# Antiviral activity of Engystol^® ^and Gripp-Heel^®^: an in-vitro assessment

**DOI:** 10.1186/1476-8518-8-6

**Published:** 2010-11-16

**Authors:** Kerstin Roeska, Bernd Seilheimer

**Affiliations:** 1 Biologische Heilmittel Heel GmbH, Baden-Baden, Germany

## Abstract

**Background:**

Infections with respiratory viruses can activate the innate immune response - an important host defence mechanism in the early stage of viral infection. Interferon (IFN) release, triggered by virus infection, is an important factor in establishing an antiviral state, where IFN activation occurs prior to the onset of the adaptive immune response.

The two ultra-low-dose combination medications, Engystol^® ^and Gripp-Heel^®^, have documented efficacy for the treatment of the respiratory infections. However, the underlying antiviral mechanisms remain elusive.

**Methods:**

It was the goal to investigate whether Engystol^® ^and Gripp-Heel^® ^display antiviral activity in a prophylactic treatment protocol (2, 24 and 48 h pre-incubation) using a plaque reduction assay and whether the medications affect the release of type 1 IFN in virus-susceptible cell lines and human peripheral blood mononuclear cells (PBMCs).

**Results:**

Both medications demonstrate prophylactic effect against viral respiratory virus replication. However, when the incubation was continued for up to 5 days, both medications exhibited a pronounced antiviral effect which was dependent on the pre-incubation time. Moreover, in co-stimulated HeLa cells as well as in activated PBMCs Gripp-Heel^® ^and Engystol^® ^demonstrated an increased type 1 IFN production.

**Conclusions:**

Engystol^® ^and Gripp-Heel^® ^inhibited the replication of a variety of respiratory viruses. Additionally, we showed that pre-incubation affects the magnitude of the inhibitory effect differently for the various tested viruses. Both medications stimulate type 1 IFN release in different cell systems which suggests that their antiviral activity may be mediated possibly via modulation of the antiviral type 1 IFN host response.

## Introduction

Viral infections of the respiratory tract are among the most common diseases for which patients seek medical advise. Some causative viruses for the common cold include influenza, parainfluenza, respiratory syncytial virus (RSV), rhinovirus (HRV) and adenovirus. The organism's immune system is normally well prepared to recognize and trigger host defence mechanisms to limit the spread of the viral infection.

The two ultra-low-dose complex medications (ULDCM) Engystol^® ^and Gripp-Heel^® ^are frequently used for prophylactic as well as acute symptomatic treatment of infectious diseases. In observational studies, Engystol^® ^showed a reliable therapeutic efficacy [1], reduced clinical symptoms and brought on more rapid relief [2]. A study comparing an Engystol^® ^treatment group with a common over-the-counter (OTC) treatment demonstrated that the efficacy of the ultra-low-dose combination medication was non inferior to the OTC treatment group [3]. However, there was a tendency for more rapid symptomatic improvement in the Engystol^®^-treated group. Gripp-Heel^® ^is mainly used symptomatically during a viral infection. An observational study showed that for the symptomatic treatment of a mild viral infection, Gripp-Heel^® ^is as effective as conventional therapies consisting of antitussives and nonsteroidal antiinflammatory drugs (NSAIDS) [4,5].

Only a limited number of studies have provided experimental evidence of how Engystol^® ^and Gripp-Heel^® ^exert their therapeutic efficacy. Nevertheless, previous *in vitro *experiments have demonstrated the antiviral activity against a broad panel of viruses. These studies showed reductions in infectivity against a panel of human respiratory viruses such as herpes simplex virus, adenovirus, influenza A virus, RSV, parainfluenza virus, rhinovirus and coxsackievirus [6,7]. Other *in vitro *studies have demonstrated that Engystol^® ^exerts modulatory effects on the immune system in terms of phagocytic activity, granulocyte function and improved humoral response [8-13]. However, the research conducted on this topic so far falls short on clarifying the possible molecular mechanisms of Engystol^® ^and Gripp-Heel^® ^in either the laboratory or the clinical setting.

The innate immune response is the first guardian in defending the body against pathogens. Central to this host antiviral response is the production of interferons (IFNs). There are two types of IFNs: type I or 'viral' IFNs (IFN-α, IFN-β and IFN-ш) and type II IFN (IFN-γ). The synthesis of type I IFN is triggered by viral infection acting on IFN-regulatory factors (IRFs), while type II IFN is induced by mitogenic or antigenic stimuli [14]. The regulation of IFN production is dependent on the virus strain, the kind of infected host cell and type of IFN. Multiple Toll-like receptors (TLR)-dependent (TLR-3,-4,-7 and -9) and independent -RIG-I (cytoplasmic helicase RNA protein) as well as Mda5 pathways are involved in the cell-type specific regulation of type I IFNs. Collaboration between the pathways is required to ensure a robust and controlled activation of antiviral response. Induction of type I IFN is regulated at the transcriptional level and is specifically achieved by members of the IRF transcription factor family [15]. Type I IFN induces various genes that encode proteins involved in innate and adaptive antiviral immune responses.

This study aimed to investigate the antiviral activity of Gripp-Heel^® ^and Engystol^® ^using a pre-treatment ("prophylactic" treatment protocol) and a continuous ("therapeutical" treatment protocol) by means of plaque reduction assay. As a second step, we wished to establish whether both preparations exert their antiviral activity by stimulating the host's IFN response. We were indeed able to demonstrate for the first time, that both medications can stimulate IFN production in an epithelial cell line as well as in cells of the immune system (PBMCs).

## Materials and methods

### Test preparations

Gripp-Heel^® ^(stock 24335-05.2011) and Engystol^® ^(stock 22981-02.2001) solution were supplied as sterile ampoules (1.1 ml H_2_O) from Biologische Heilmittel Heel (Baden-Baden, Germany). Gripp-Heel^® ^contains *Aconitum *(D4), *Byronia *(D3), *Lachesis *(D11), *Eupatorium perfoliatum *(D2) and phosphorus (D4). Engystol^® ^contains *Vincetoxicum hirundinaria *(D6), *Vincetoxicum hirundinaria *(D10), *Vincetoxicum hirundinaria *(D30), sulphur (D4) and sulphur (D10). The test preparations were diluted in cell culture medium before addition to the cell culture. Final concentrations in the assays ranged from 1:4 to 1:320.

### Cell culture and viruses

Human rhinovirus B serotype 14 (HRV-14) was obtained from the Institute for Virology (University of Jena, Germany). Influenza A virus (FluA), Chile 1/83 (H1N1), herpes simplex virus 1 (HSV-1, strain Thea), vesicular stomatitis virus (VSV, FL1), respiratory syncytial virus (RSV, strain Long), parainfluenza type 3 (Para3) and adenovirus type 5 (Ad5) were obtained from the Friedrich-Löffler Institute (FLI) Tübingen and the Department of Medical Virology and Epidemiology of Virus Diseases of the Hygiene Institute at University of Tübingen, respectively.

#### For IFN assays

HeLa cells (University of Jena, Germany) were incubated with UV-inactivated HRV-14, Hep-2 cells (CCL-23, ATCC) with UV-inactivated HSV-1 and Madin-Darby canine kidney cells (MDCK) with UV-inactivated FluA.

#### For cultivation of viruses

Hep-2 and HeLa cells were cultivated in MEM with Hanks' buffered saline solution containing 2% fetal calf serum (FCS, PAA, Pasching, Austria), 25 mM MgCl_2_, 2 mM L-glutamine, 100 U/ml penicillin and 0.1 mg/ml streptomycin). Cells were incubated in serum-free MEM containing 1 μg/ml trypsine, 2 mM L-glutamine, 100 U/ml penicillin and 0.1 mg/ml streptomycin.

#### Determination of virus titre

The respective cells were incubated in 12-or 24 well tissue culture dishes with serially diluted serum-free virus stock solutions for 1 h at 34°C as described elsewhere [6].

### Isolation of PBMCs

Peripheral blood mononuclear cells (PBMCs) were isolated from healthy donors (Wiener Rotes Kreuz, Wien, Austria). Healthy donors were identified by diagnostic parameters (negative haemogram, infection serology). The blood samples were treated with heparin (Sigma) and subjected to Ficoll-Hypaque (1,077 g/l PAA) density gradient centrifugation (30 min. 2100 rpm). The PBMCs were isolated from the interphase, subsequently washed twice with medium (RPMI 1640, 10% FCS) and counted using the trypan blue exclusion test.

The cells were incubated for 2 to 6 days with the test preparations before being tested for IFN release (96-well round bottom microtiter plates, Greiner, Bio-one, Kremsmünster, Austria, 2 × 10^5 ^cells/well, RPMI 1640, 10% FCS). In an alternative approach, cells were co-stimulated with HSV-UV to induce IFN synthesis.

### Virus titration for costimulation experiments

To induce IFN release in PBMCs and virus-susceptible cell lines, virus suspensions of inactivated HSV-1, HRV-14 and FluA were employed.

High titre virus suspensions (1 × 10^8 ^PFU/ml) of the respective viruses were produced and inactivated with an UV-GS linker. The virus titre inducing 20-30% IFN synthesis (calculated with an IFN-α standard) was determined in serial dilutions and kinetic experiments.

### Plaque formation assays and virus-specific ELISA

The antiviral activity of Gripp-Heel^® ^against influenza, Para3, RSV, HRV-14, HSV-1 and Ad5 was determined by means of a plaque formation assay or cytopathogenic effect (CPE), respectively as described elsewhere [6]. For Ad5 a virus specific ELISA was employed. Briefly, the virus permissive cell lines were incubated with the test medications at different concentrations for 2h, 24h and 48h. The test medication was removed and the cells were infected with a multiplicity of infection (MOI) of 0.00037 (Flu A), 0.00044 (Para3), 0.00046 RSV, 0.00042 (HRV-14), 0.0004 (HSV-1) and 0.004 (Ad5). The virus inoculum was removed and subsequently 1) cells were overlaid with solid medium only ("prophylactic" protocol) or 2) cells were overlaid with solid medium containing the test substances ("therapeutic" protocol) and cultivated until in the control plaques or CPE appeared. The percentage of inhibition was calculated in reference to the untreated control (100% inhibition) and expressed as relative inhibition (n = 2 in duplicates).

### Interferon-α specific ELISA

A commercial IFN-α ELISA (Biosource) was used to determine the quantity of IFN-α in the cell culture supernatant. Briefly, cells (96-well round bottom microtiter plates, Greiner, Bio-one, Kremsmünster, Austria, 2 × 10^5 ^cells/well, RPMI 1640, 10% FCS) were seeded and incubated with the test preparations for the indicated time points. Additional samples were co-stimulated with the appropriate UV-inactivated virus. After incubation for the indicated time points, the supernatant was collected and the IFN-α content was quantified according to the manufacturer instructions. The quantity of IFN-α is proportional to the extinction values at OD450 nm and is calculated in pg/ml using an IFN-α standard curve. To calculate an increase or decrease in percentage terms, the MEM control or co-stimulated control, respectively, was defined as 100% IFN-α and thereby served as relative values compared to control. The data represent the mean values +/- SD (n = 3).

## Results

### 1. Antiviral effect of Gripp-Heel^® ^and Engystol^®^

As a first step we asked whether Gripp-Heel^® ^and Engystol^® ^reduce infectivity in the prophylactic setting where cells are only exposed to the medications previously to infection. The permissive cell lines which are susceptible only for a certain virus (see below) were thereby pre-treated for 2 h, 24 h and 48 h with the test preparations, washed and subsequently infected with the different viruses (HRV-14, HSV-1, FluA, Para 3, RSV and Ad5). In the plaque reduction assays and a virus-specific ELISA (Ad5), Engystol^® ^(ES) and Gripp-Heel^® ^(GH) induced a slight prophylactic virus inhibition at the lowest dilution (1:5) when pre-incubated for 48 h against FluA [ES: 20.7%; GH:15,4], HRV-14 [ES: 25.9%; GH: 18.8%] and HSV-1 [ES:19.6%; GH: 15.9%]). However, a dose-response relationship was not observed and also shorter incubation times showed no effect (data not shown).

**Figure 1:**
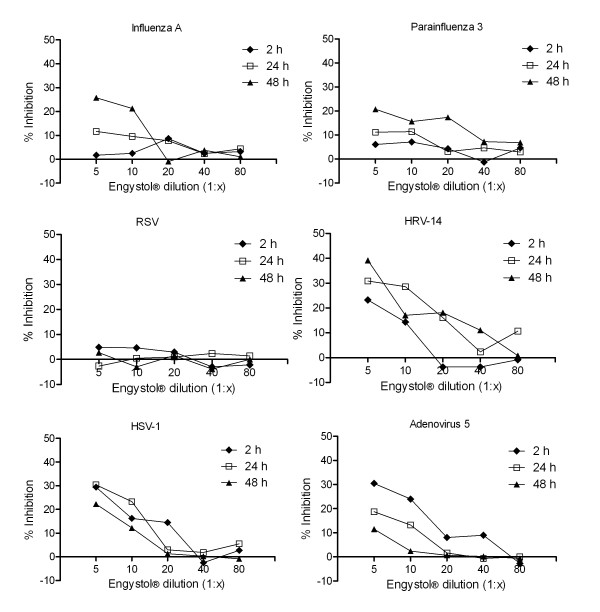
**Antiviral effect of Engystol^® ^against several viruses**. Inhibitory effect of Engystol^® ^on Influenza A (a), Parainfluenza 3 (b), RSV (c), HRV-14 (d), HSV-1 (e) and Adenovirus 5 (f) were determined using plaque reduction assay and virus-specific ELISA for Ad5, respectively ("therapeutic approach"). Data are shown as percentage of inhibition compared to the untreated control (100% inhibition, n = 4).

**Figure 2:**
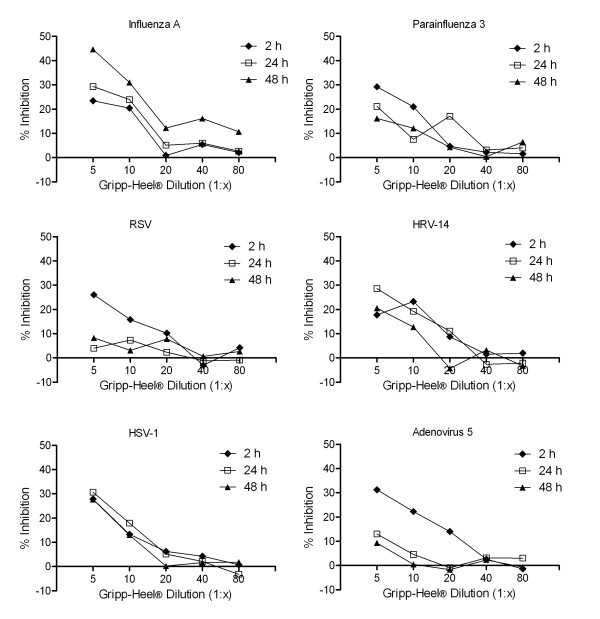
**Antiviral effect of Gripp-Heel^® ^against several viruses**. Inhibitory effect of Gripp Heel^® ^on Influenza A (a), Parainfluenza 3 (b), RSV (c), HRV-14 (d), HSV-1 (e) and Adenovirus 5 (f) determined using plaque reduction assay and virus-specific ELISA for Ad5, respectively ("therapeutic approach"). Data are shown as percentage of inhibition compared to the untreated control (100% inhibition, n = 4).

In contrast, when the cells were continued to be incubated in the presence of test preparations, we observed a dose-dependent reduction of infectivity ranging from 20 to 44%, depending on the virus and pre-incubation time (Fig 1, 2). In particular, Gripp-Heel^® ^and Engystol^® ^showed dose-dependent antiviral activity against FluA, HRV-14, HSV-1, Ad5 and Para 3, with Engystol^® ^(Figure 1a-f, 2a-f) having only a moderate effect on Para 3 (20.7% at dilution 1:5). In contrast, a dose-dependent inhibition of viral replication of RSV was noted for Gripp-Heel^®^, but not for Engystol^®^. The observed inhibitory effects were influenced by the pre-incubation time chosen. In the case of FluA, both preparations showed highest efficacy when pre-incubated for 48 h (ES: 25.7% and GH: 44.6% at 1:5 dilution). For HSV-1, the best effects were observed after 24 h of pre-incubation (ES: 30.4%; GH: 30.7% at 1:5 dilutions). For Ad5, the highest inhibition was observed at 2 h (ES: 30.5%; GH: 31.3% at 1:5 dilution), and for HRV-14, the longer pre-incubations (24 h and 48 h) showed best efficacy (ES: 39.2%; GH: 28.6% at 1:5 dilution). Antiviral activity of Gripp-Heel^® ^against RSV was found only after 2 h pre-incubation (26.1%); also for Para 3, the best inhibitory effect was determined after 2 h (29.2%). Through our evaluation of the prophylactic activity, we observed that the pre-incubation time influences the magnitude of the antiviral effect in a virus specific manner when the treatment with test preparation was continuous.

### 2. Effect of Engystol^® ^on type 1 IFN production in virus-susceptible cell lines

To investigate whether Engystol^® ^can evoke type 1 IFN production without previous exposure to viral structures, the effect on virus susceptible cell lines were tested, which were incubated with the medication in five dilutions for two days. We observed no significant increase in IFN (IFN-α ELISA and bioassay) in the unstimulated cell lines in the presence of Engystol^® ^(data not shown), indicating that Engystol^® ^could not induce a spontaneous IFN release in the tested cell lines.

Therefore, we expanded our experimental set-up to determine whether Engystol^® ^might exert its effect in virus-activated cells. To this purpose HeLa, Hep-2 and MDCK cell lines were incubated with the corresponding UV-inactivated virus (described in method section) and cultivated in the presence of Engystol^® ^(or MEM control, respectively) for 2 days.

**Figure 3:**
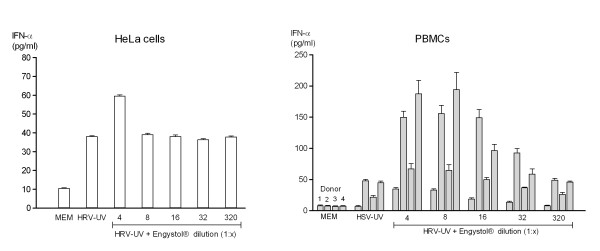
**In-vitro type 1 IFN production following Engystol^® ^incubation**. Effect of Engystol^® ^on type 1 IFN production in HRV-UV infected HeLa (a) and HSV-UV-co-stimulated PBMCs (b) was determined using IFN-α specific ELISA 5 days after incubation. In figure a data represent mean values ± SD (N = 3). For the PBMC setting 4 different donors were used for each treatment group (first column = donor 1, second column = donor 2, third column = donor 3, fourth column = donor 4).

No increase in IFN release was observed for MDCK cells infected with FluA or Hep-2 cells infected with HSV-1 (data not shown). However, in HeLa cells infected with inactivated HRV an increase in IFN production was obtained in both assays. A 56%-increase in IFN release compared to control was observed at a 1:4 dilution of Engystol^® ^in the ELISA assay (Figure 3a).

### 3. Effect of Engystol^® ^on type I IFN production in PBMCs

In order to determine whether Engystol^® ^modulates the IFN release in cells of the immune system, human peripheral blood mononuclear cells (PBMCs) were isolated from 4 healthy donors and incubated for either 2 or 5 days with 5 dilutions of Engystol^®^. In 3 of the 4 donors, a spontaneous, although very small IFN release (< 15 pg/ml) was noted (data not shown). In contrast, when the IFN response was primed with UV-inactivated HSV-1 Engystol^® ^elicited a pronounced increase in IFN after 2 days and 5 days of co-cultivation. In the ELISA an up to 4-fold increase in IFN-α production was observed at a 1:4 dilution at day 5 of incubation (Figure 3b). Even higher dilutions showed a modest dose-response relationship. These results were confirmed in an independent set of experiments with 3 additional healthy donors (data not shown).

### 4. Effect of Gripp-Heel^® ^on type 1 IFN production in virus-susceptible cell lines

**Figure 4:**
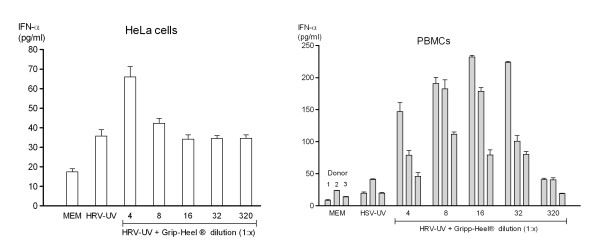
**In-vitro type 1 IFN production following Gripp-Heel^® ^incubation**. Effect of Gripp-Heel^® ^on type 1 IFN production in HRV-UV infected HeLa (a) and HSV-UV-co-stimulated PBMCs (b) was determined using IFN-α specific ELISA 4 days after incubation. In figure a data represent mean values ± SD (N = 3). For the PBMC setting (b) 4 different donors were used for each treatment group (first column = donor 1, second column = donor 2, third column = donor 3, fourth column = donor 4).

According to Engystol^® ^the effect of Gripp-Heel^® ^on IFN release was determined. No significant increase in IFN was observed in the unstimulated cell lines MDCK, Hep2 and HeLa in the presence of Gripp-Heel^® ^(data not shown). Also, Gripp-Heel^® ^did not affect the IFN release in virus-activated MDCK (FluA) and Hep2 (HSV-1) cells (data not shown). However, in HeLa cells treated with inactivated HRV Gripp-Heel^® ^displayed a modest dose-dependent effect on IFN-α production as quantified by ELISA (Figure 4a).

### 5. Effect of Gripp-Heel^® ^on type 1 IFN production in PBMC

Finally, the effect of Gripp-Heel^® ^on the IFN levels in stimulated and unstimulated PBMCs was determined. In a first set of experiments, cells obtained from healthy donors were incubated with Gripp-Heel^® ^for 4 days. Blood cells from one (donor 1) of 3 donors exhibited a small but notable spontaneous release in IFN (data not shown). In the HSV-primed cells a 4 day incubation with Gripp-Heel^® ^induced IFN release in all three blood samples, as quantified by ELISA (Figure 4b). Notably, the blood from donor 1 showed a pronounced IFN release even at a higher dilution of Gripp-Heel^® ^(1:32). This finding was confirmed by similar results from a second set of experiments (data not shown).

## Discussion

This study provides the first results towards understanding how the two ultra-low-dose combination medications Engystol^® ^and Gripp-Heel^® ^may possibly exert their antiviral effect, as documented in observational studies and in a small number of experimental studies [6,7]. Employing virus-permissive cell lines, we confirmed the inhibitory effect of both medications against a broad panel of respiratory viruses *in vitro *(plaque assay) and provide the first evidences, using two different experimental settings (cell lines and PBMCs) which suggest that the antiviral effect may be due to the triggered increase in type 1 IFN production.

Engystol^® ^and Gripp-Heel^® ^are used prophylactically to ward off viral infections and are valued for their good tolerability and lack of any known adverse effects [2,5]. The established clinical efficacy, yet not clarified mechanism of action of the two preparations, formed the objective of our studies here to investigate the ability of these medications to reduce infectivity under *in vitro *conditions. We found that both preparations had a modest prophylactic effect against infection with HSV, FluA and HRV, whilst Engystol^® ^was slightly more effective than Gripp-Heel^® ^in this case. When the incubation was continued over the course of the experiment ("therapeutic" setting), however, both medications showed a pronounced inhibitory activity against all six tested respiratory viruses except for RSV, which was not inhibited by Engystol^®^. These findings agree with previously published data, which demonstrated similar results for Gripp-Heel^® ^against a broad panel of RNA and DNA viruses [6].

For Engystol^®^, the results from the plaque formation assays, confirm in part previously published results [7]. We did not observe such a strong inhibition (70% to 80%) of Ad5 and HSV, although it should be noted that these high values were achieved with the highest concentration (1:2). As we observed toxicity at a dilution of 1:2 (data not shown), we applied higher dilutions (>1:5). We could not confirm the reported inhibitory effect against RSV (37% at dilution 1:2), but did observe an inhibitory effect against FluA. These different findings may be due to different multiplicity of infections (MOIs) employed and dilutions of the test preparation. Despite our results as well as the previous studies suggest that the clinical benefits of both preparations may be due to inhibition of virus infectivity. Oberbaum and colleagues suggested that Engystol^® ^might have a direct effect on virus replication, since they did not observe any induction of IFN-α in an epithelia and embryonic fibroblast cell line [7]. As discussed below induction of IFN in cell lines is highly virus- and cell type dependent and might critically depend on the virus titre. Since Engystol^® ^showed efficacy against a broad panel of structurally different viruses such as RNA and DNA viruses - enveloped and non-enveloped -, it seems likely that Engystol^® ^and Gripp-Heel^® ^also modulate host defence mechanisms.

Type 1 IFN production plays an important role in antiviral response and involves a large family of multifunctional immuno regulatory proteins. IFN-α/β is induced by virus infection and cells that respond to IFN establish an antiviral state. These statements are based on the observation that IFN-α/β receptor knock out does not result in an antiviral state and strongly indicates that IFN cytokines are of particular importance for the immune response to viral pathogens [16]. Induction of type I interferon can be mediated via various cellular pathways. The "classical" pathway is characterized by the phosphorylation of IFN regulatory factors (IRFs) [17,18]. Furthermore, IFN type I can be induced via Toll-like receptor (TLR) signalling, modulating the development of innate and adaptive immune systems [17,18]. Apparently, the induction of IFN type I molecules results from activation of distinct interacting pathways dependent on pathogenic factors and host cell determinants. We postulate that the observed antiviral effect of Engystol^® ^and Gripp-Heel^® ^might involve stimulation of IFN.

In the current study two RNA viruses - FluA and HRV - as well as the DNA virus HSV were tested on susceptible cell lines for their potential to induce type I IFN production. However, only HRV could induce type I IFN production in epithelial HeLa cells. In epithelial cells IRF-3, but not IRF-7, is constitutively expressed and controls IFN-α/β induction, as described above [19]. Therefore, IFNα/β gene induction occurs sequentially, wherein the initial IFN-β induction by IRF-3 (first phase) triggers a positive feedback loop via IRF-7 induction and IFN-α production, thus amplifying the response [17]. Adding to the complexity of these processes, IFN is induced by cytoplasmic RNA [20,21]. Thus, it may be possible that cytosolic dsRNA, which is normally produced in the course of virus replication, would have been required to induce IFN response and which might account for the failure in IFN production for FluA and HSV. In the case of HRV, possibly a different mechanism might account for the observed IFN production.

This study shows for the first time using an IFN-α specific ELISA and a functional bioassay that both medications increase IFN production in PBMCs, in which type I IFN production was triggered using UV-inactivated HSV. Inactivated HSV has been previously shown to induce type I IFN in primary human cells [22,23]. In particular, IFN-α production can be induced in human mononuclear cells in the presence of purified, recombinant HSV-1 glycoprotein D [24,25].

With regard to the cell types responsible for the IFN type I release it is noted, that almost any nucleated cell in culture, such as macrophages or lymphocytes, can produce IFNs. However, precursor dendritic cells with plasmacytoid morphology (pDC) were characterized as a specialized subset of cells producing the bulk amount of IFN type one during viral infections [26,27]. It is conceivable that pDCs may account for the large part of the IFN response in isolated PBMCs reported here. Both Gripp-Heel^® ^and Engystol^® ^triggered an increase in IFN production only in the presence of viral structures, suggesting that Engystol^® ^and Gripp-Heel^® ^rather exert their effect when the immune system is already active than during healthy and inactive immune conditions. We speculate that this subpopulation may be the target for the IFN release induced by Gripp-Heel^® ^and Engystol^®^. However, this needs to be scientifically proven in further experiments.

IFN-γ plays a major immunomodulatory role and is a key mediator of virus-specific cellular immunity. IFN-α/β can promote IFN-γ expression in T-cells [28] and appears to play a key role in the coordination of innate and adaptive immune response during viral infection. In this respect, a recent report from Engbergs and colleagues is noteworthy, as these authors suggest that Engystol^® ^may increase the percentage of IFN-γ-producing lymphocytes *in vitro *[8]. During the adaptive phase, IFN-γ is mainly produced by activated T-cells, whilst in the innate phase, natural killer cells are thought to be the main source of the newly formed IFN-γ [18].

## Conclusion

In summary, the data presented here provide further evidence that the two medications, Gripp-Heel^® ^and Engystol^®^, have antiviral effect *in vitro*. Our studies show for the first time that Engystol^® ^and Gripp-Heel^® ^increase IFN release in HRV-activated HeLa cells and induce type 1 IFN release in primary immune cells primed with replication-deficient HSV. We suggest that these preparations exert their antiviral effect by modulating the type 1 IFN response. Further studies to elucidate the signalling pathways involved are warranted in order to also establish whether pDCs are indeed the main target cells of these medications. Focusing on dendritic cells could be especially useful, as these cells initiate T-cell response and may be the link between the innate and adaptive immune response.

## Competing interests

The complex combination preparations Engystol and Gripp Heel^® ^are commercialised products of Biologische Heilmittel Heel GmbH, Baden-Baden, Germany. The authors are employees of Biologische Heilmittel Heel GmbH.

## Authors' contributions

BS and KR have substantially contributed to the interpretation of the data and were highly involved in drafting and revising the manuscript. BS has given final approval of the version to be published.
